# A review of historical trends in *Anopheles gambiae* Giles (Diptera: Culicidae) complex composition, collection trends and environmental effects from 2009 to 2021 in Mpumalanga province, South Africa

**DOI:** 10.1111/mve.12761

**Published:** 2024-09-05

**Authors:** Kayla P. Noeth, Maria L. Kaiser, Thabo Mashatola, Yael L. Dahan‐Moss, P. Avhatakali Matamba, Belinda Spillings, Riann Christian, Erika Erlank, B. Power Tshikae, Eunice Jamesboy, Silindile Sibambo, Busisiwe G. Nkosi, Brian T. Silawu, Lazarus J. Mkhabela, Fanuel S. Ndlovu, Thembekile P. Mgwenya, Maureen Coetzee, Basil D. Brooke, Lizette L. Koekemoer, Givemore Munhenga, Shüné V. Oliver

**Affiliations:** ^1^ Centre for Emerging Zoonotic & Parasitic Diseases National Institute of Communicable Diseases Johannesburg South Africa; ^2^ Wits Research Institute for Malaria, Faculty of Health Sciences University of the Witwatersrand Johannesburg South Africa; ^3^ Malaria Elimination Programme, Mpumalanga Department of Health Nelspruit South Africa

**Keywords:** *Anopheles arabiensis*, *Anopheles merus*, bionomics, surveillance

## Abstract

South Africa is a frontline country for malaria elimination in the southern African region. It has three malaria‐endemic provinces, each with its own transmission pattern. The elimination of malaria depends, in part, on controlling and/or eliminating vectors responsible for transmission. Sustained entomological surveillance is an important factor to consider when shifting from a control to elimination framework. The Ehlanzeni district in Mpumalanga province is a key entomological sentinel surveillance area. It is one of the malaria‐endemic districts in South Africa with higher rates of malaria incidences. As such, entomological data about the *Anopheles gambiae* Giles (Diptera: Culicidae) complex have been collected in this province over a substantial period. These data are stored in a pre‐existing institutional database. An analysis of the trends that can be observed from this database has not been performed before. This retrospective (longitudinal) analysis provides a summary of the *An. gambiae* complex vector composition in this region from 2009 to 2021. Routine surveillance data were correlated with climatic data (obtained from the NASA LaRC POWER project database) for the same period to assess the role of climatic factors in vector dynamics. This review also identifies a number of limitations in the data collection process across the sampling period and provides recommendations on how to strengthen the database going forward. The most abundant member of the *An. gambiae* complex since 2009 in the province was *An. merus* Dönitz followed by *An. arabiensis* Patton. Collection methods used showed that human landing catches were successful for collecting *An. arabiensis*, while pit traps were the most effective in collecting *An. merus* and *An. quadriannulatus* Theobald. The latter two species were mainly collected in spring, whereas *An. arabiensis* abundance was larger during autumn collections. Vector abundance was not significantly correlated with annual climatic data. The information gained from this database provides insights into the vector dynamics of the Ehlanzeni district of the Mpumalanga province.

## Graphical abstract

- *Anopheles* merus was the most abundant *Anopheles* species in Mpumalanga between 2005 and 2020.
- Lack of correlation between vector abundance and macroclimatic variables suggests microclimate plays an important role in observed vector abundance trends.
- Collection data suggest tailor‐made control efforts may be more effective in capturing exophilic species and reducing residual malaria transmission in Mpumalanga.

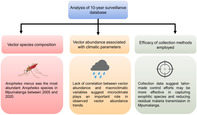

## INTRODUCTION

Malaria is the most significant vector‐borne disease in the world (World Health Organization, 2017). In South Africa, malaria has been a consistent public health threat since the early 1900s, being endemic in three provinces viz. Limpopo, Mpumalanga and KwaZulu‐Natal (Coetzee, Kruger, et al., 2013). While significant efforts have been made to control malaria, local transmission of the disease still occurs presenting low‐to‐moderate risk (Moonasar et al., 2021). This low level and often outdoor transmission is known as residual malaria (Carnevale & Manguin, 2021). This threatens South Africa's malaria elimination agenda, which aims to achieve interruption of transmission and prevent re‐establishment (National Department of Health, 2019).

The transmission of malaria depends on several factors. The entomological factors include vector species composition, abundance, breeding site characteristics, vector behavioural traits (e.g., feeding, resting and host preference behaviours) and insecticide resistance (Durnez & Coosemans, 2013; Ferguson et al., 2010; Russell et al., 2013; Sherrard‐Smith et al., 2019). A variety of environmental factors are also involved. These include climatic factors such as ambient temperature, rainfall patterns and relative humidity (Adeola et al., 2017; Carnevale & Manguin, 2021). A clear understanding of these factors will assist in devising effective control strategies for the elimination or sufficient control of vector populations, which will in turn assist with achieving and maintaining malaria elimination. This is especially critical on a region‐by‐region basis, since vector bionomics have been shown to differ from one region to the next (Durnez & Coosemans, 2013).

Members of the *An. gambiae* Giles (Diptera: Culicidae) complex are some of the most abundant vectors in South Africa, along with the *An. funestus* Giles group. The *An. gambiae* complex consists of 10 species (Barrón et al., 2019; Coetzee, Hunt, et al., 2013; Mattingly, 1977; Tennessen et al., 2021; White, 1974, 1985). In South Africa only *An. arabiensis* Patton, *An. quadriannulatus* Theobald and *An. merus* Dönitz occur (Gillies & Coetzee, 1987; Spielman, 1970). Of these, only *An. arabiensis* has been directly implicated locally as a vector although it is highly likely that *An. merus* also contributes to local transmission (Govere et al., 2000; Mbokazi et al., 2018). Their ecological and biological characteristics vary between, and in some cases, within species (Gillies & Coetzee, 1987, Spielman, 1970), which poses a considerable challenge to understanding the bionomics of malaria vectors in South Africa. This involves understanding the role of secondary vector species, factors affecting life history traits (e.g., pollution), the impact of insecticide resistance on control interventions, and the feeding‐, resting‐, and host selection preferences of vectors.

Regular surveillance is critical to address these questions. While annual surveillance can give a snapshot of the situation in that year, an overview of multiple years of data can offer insight into bionomical trends over time. This information can assist in making evidence‐based decisions for vector control interventions.

The Ehlanzeni district in Mpumalanga province is one of the malaria‐endemic districts in South Africa affected with higher rates of malaria incidence compared with other malaria‐endemic districts in KwaZulu‐Natal and Limpopo (Raman et al., 2016). This district is a key surveillance area and has been the focus of surveillance for over a decade. As such, a large body of entomological data has been collected in this area, especially on the *An. gambiae* complex. Despite the surveillance programme being a government initiative with limited resources, funding, and standardised data collection methods, the accumulated data may still provide valuable insights into vector behaviour. Additionally, the database could help identify gaps in the data collection process.

In this review, surveillance data collected between 2009 and 2021 in Ehlanzeni district was analysed to provide insight into the historical trends of vector density and the potential impact of climate. Recommendations for improving the database were also investigated and have been provided.

## MATERIALS AND METHODS

### Overview

The dataset summarised in this study is curated by the Vector Control Reference Laboratory of the National Institute for Communicable Diseases in Johannesburg, South Africa. The data was collected in collaboration with the National Malaria Control Programme of the National Department of Health of South Africa or as part of past research projects. Data for the period 2009–2021 were analysed and summarised.

### Mosquito collection and identification methods

Adult *Anopheles* mosquitoes were captured using standard collection methods. These included human landing catches, natural resting shelters, man‐made pit traps, indoor‐ and outdoor placed clay pots, modified plastic buckets and carbon dioxide (CO_2_) baited tent traps (Braack et al., 2020; Dandalo et al., 2017; Muirhead Thomson, 1951; Muirhead‐Thomson, 1958). Other methods included specimen collections from disused tyres, window traps and pit toilets (Dandalo et al., 2017). The immature stages were collected as larvae and pupae from natural pools, dams, stagnant ponds, water‐filled potholes and other sources of still/standing water (Mbokazi et al., 2018).

Adult specimens were preserved on silica in 1.5‐mL reaction tubes and were morphologically identified using external morphological characteristics (Coetzee, 2020; Gillies & Coetzee, 1987). Members of the *An. gambiae* complex were identified to species using the polymerase chain reaction (PCR) assay described by Scott et al. (1993).

Larval specimens were reared to adulthood and the subsequent generation of adults were identified to species as described for adult collections.

### Data collection and organisation

The data from collections were recorded on standardised species identification sheets. The information recorded for each specimen included the unique identification number, identification result from the PCR assay, the date of collection, location of the collection, sex and the collection method. This information was collated in a single datasheet with 10,576 entries.

The data were subsequently cleaned by standardising the date format and spelling of location entries. Duplicate entries and entries without identification, date or location data were removed. This database with 7869 entries remaining was then enhanced by adding additional columns for each entry for ease of analysis. The date column (date of specimen collection) was expanded to include columns for month and year. A season column was added with the following variables: summer (01 December–29 February), autumn (01 March–31 May), winter (01 June–31 August) and spring (01 September–30 November). A municipality column was added which divided collection localities into one of three districts, viz Nkomazi, Bushbuckridge or Mbombela. Finally, a count column was added for each entry to convert the qualitative identification value to a numerical one.

### Specimen collection sites

**FIGURE 1:**
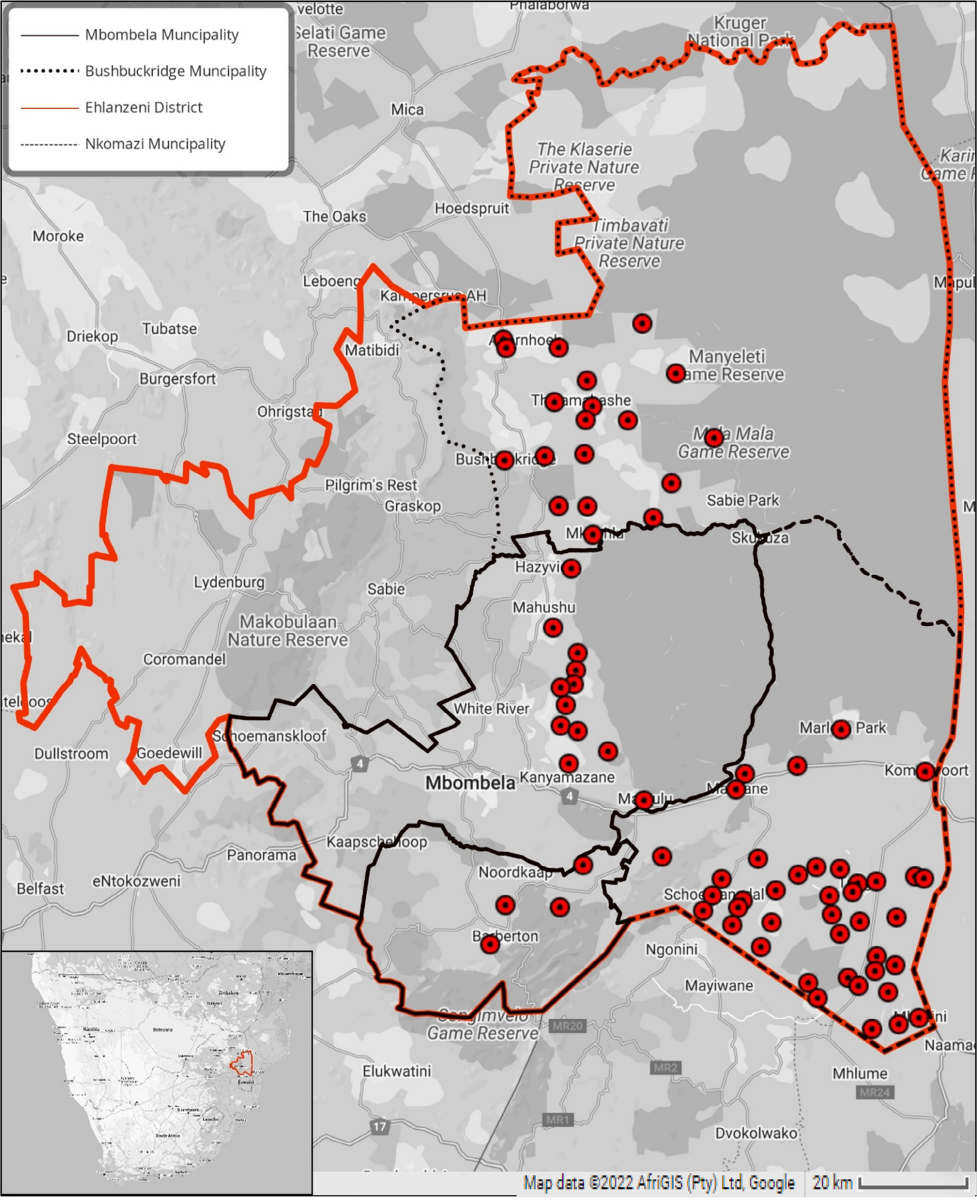
Collection sites in the surveillance areas of the Ehlanzeni district (bordered in red). The 75 sentinel sites within the Ehlanzeni district from which larval and/or adult *Anopheles* specimens were collected from 2009 to 2021 are indicated by red dots. The Nkomazi, Bushbuckridge and Mbombela municipalities are bordered in black.

*Anopheles* mosquitoes were collected from 71 sentinel sites in three different municipalities in the larger Ehlanzeni district (Figure 1), viz., Nkomazi Municipality (*n* = 40 sites), Bushbuckridge Municipality (*n* = 19 sites) and Mbombela Municipality (*n* = 12 sites).

### Data analysis

Sampling effort was defined as the number of collection days. A Spearman's rank correlation test was used to assess the correlation between sampling effort per year and the number of mosquitoes captured per year.

The total number of *An. gambiae* sensu *latu* (*s.l*.) mosquitoes collected were summarised for the entire collection period (2009–2021) by species. Sampling effort was determined for (1) each year across the sampling period, (2) each season across the sampling period, (3) each collection method across the sampling period and (4) each locality sampled across the sampling period. The number of *An. gambiae s.l*. captured per species was averaged for each year and divided by the sampling effort of each year, season and collection method.

Additionally, *An. gambiae s.l*. species density was correlated with the average temperature, precipitation and relative humidity for the Nkomazi and Bushbuckridge municipalities, as the data for these were more comprehensive than Mbombela Municipality. The total number of mosquitoes captured in each district was summed for each year across the collection period (13 years). This was correlated to the average temperature, precipitation and relative humidity for each year across the collection period. Similarly, the total number of mosquitoes were summed for each month across the collection period (i.e. all mosquitoes for the month of January across the 13‐year collection period) and correlated with climatic parameters that were likewise averaged. A more microclimatic approach was investigated by focusing on a single site, Block A, in the Nkomazi Municipality and looking at daily climatic parameters. The total number of mosquitoes captured at Block A in Nkomazi for each day of collection was correlated with climatic data from the day of collection. The significance of the results was assessed using Spearman's Rank Correlation test (Spearman, 1904). Climatic data were obtained from the National Aeronautics and Space Administration (NASA) Langley Research Centre (LaRC) POWER Project, which is funded through the NASA Earth Science/Applied Science Program (NASA Langley Research Center Atmospheric Science Data Center, 2022).

The number of *An. gambiae s.l*. mosquitoes was averaged for each sampling locality and divided by the sampling effort of each sampling locality. Species richness (number of unique species per site, abbreviated ‘R’) was determined for each sampling locality and averaged for each sampling district. The following alpha‐diversity indices were also calculated: Shannon‐Weiner Diversity Index, Simpson's Diversity Index and the Pielou's Evenness Index. The Simpson Diversity Index (abbreviated ‘D’; Equation 1) estimates species diversity within a community that accounts for both the number of unique species present as well as the relative abundance of each species present. The Shannon Diversity Index (abbreviated ‘H’; Equation 2) estimates the species diversity within a community, taking into account the number of unique species present. The Pielou Evenness Index (abbreviated ‘J’; Equation 3) measures whether species within a community are equally abundant (i.e., even). Equations are available in Figure S1. A Shapiro–Wilk (Shapiro & Wilk, 1965) test showed that the data for each alpha diversity index were not normally distributed. A Kruskal–Wallis test (Kruskal & Wallis, 1952) was performed to assess the differences between districts for each index.

All statistical analyses were performed in R (R Core Team, 2023).

## RESULTS

### Sampling effort

**FIGURE 2:**
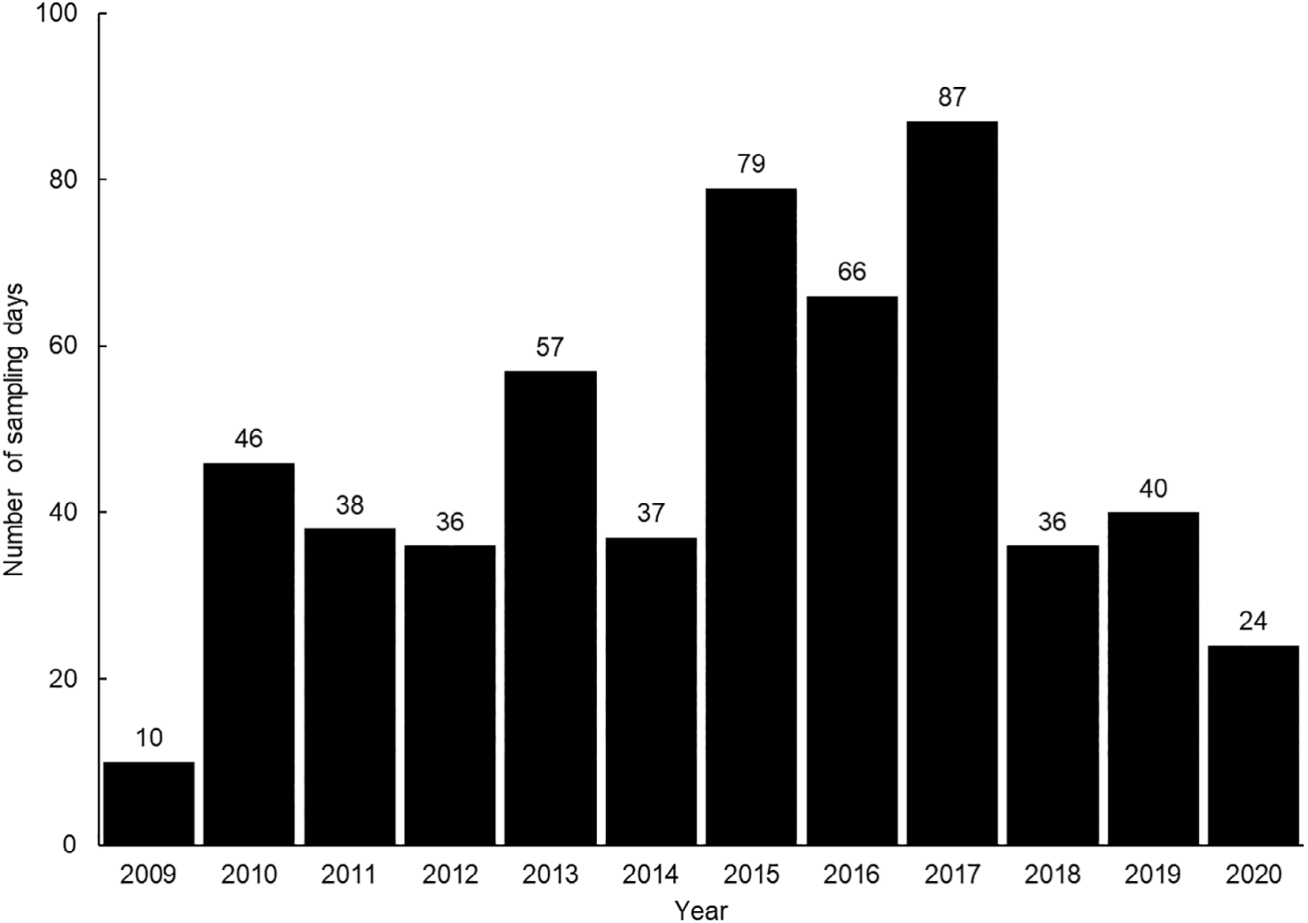
Sampling effort (defined here as the number of collection days) by year across the sampling period.

Sampling effort was variable across the surveillance period, with the most sampling days occurring in 2017 (*n* = 87) and the least in 2009 (*n* = 10) (Figure 2). A Spearman's rank correlation test showed a significant positive correlation between sampling effort (number of collection days) and mosquito abundance (*r*_*s*_ = 0.82, *p* = 0.0011).

### Total mosquitoes collected between 2009 and 2021

The total number of *An. gambiae* complex species collected and identified between 2009 to 2021 in Mpumalanga province was 7812. *Anopheles merus* accounted for 50.3% of collections (*n* = 3933), while *An. quadriannulatus* and *An. arabiensis* contributed 30.1% (*n* = 2348) and 19.6% (*n* = 1531), respectively.

### Total mosquitoes collected per season

*Anopheles merus* and *An. quadriannulatus* were most abundant during spring (*n* = 9.84 captures per day and *n* = 5.33 captures per day, respectively), while *An. arabiensis* was most abundant during autumn (*n* = 3.73 captures per day) (Table 1). Mosquito abundance was lowest during spring for all *An. arabiensis*, autumn for *An. merus* and summer for *An. quadriannulatus* (Table 1).

**TABLE 1:** The number of *Anopheles arabiensis*, *An. merus* and *An. quadriannulatus* captured per day for each season from 2009 to 2021 in the Ehlanzeni district of Mpumalanga province, South Africa.

	*An. arabiensis*	*An. merus*	*An. quadriannulatus*	Total
Autumn	3.73	4.88	3.96	12.57
Spring	1.48	9.84	5.33	16.64
Summer	3.28	6.51	3.44	13.23
Winter	1.86	7.41	4.34	13.62
Total	10.35	28.64	17.07	56.06

### Total mosquitoes collected per year

**FIGURE 3:**
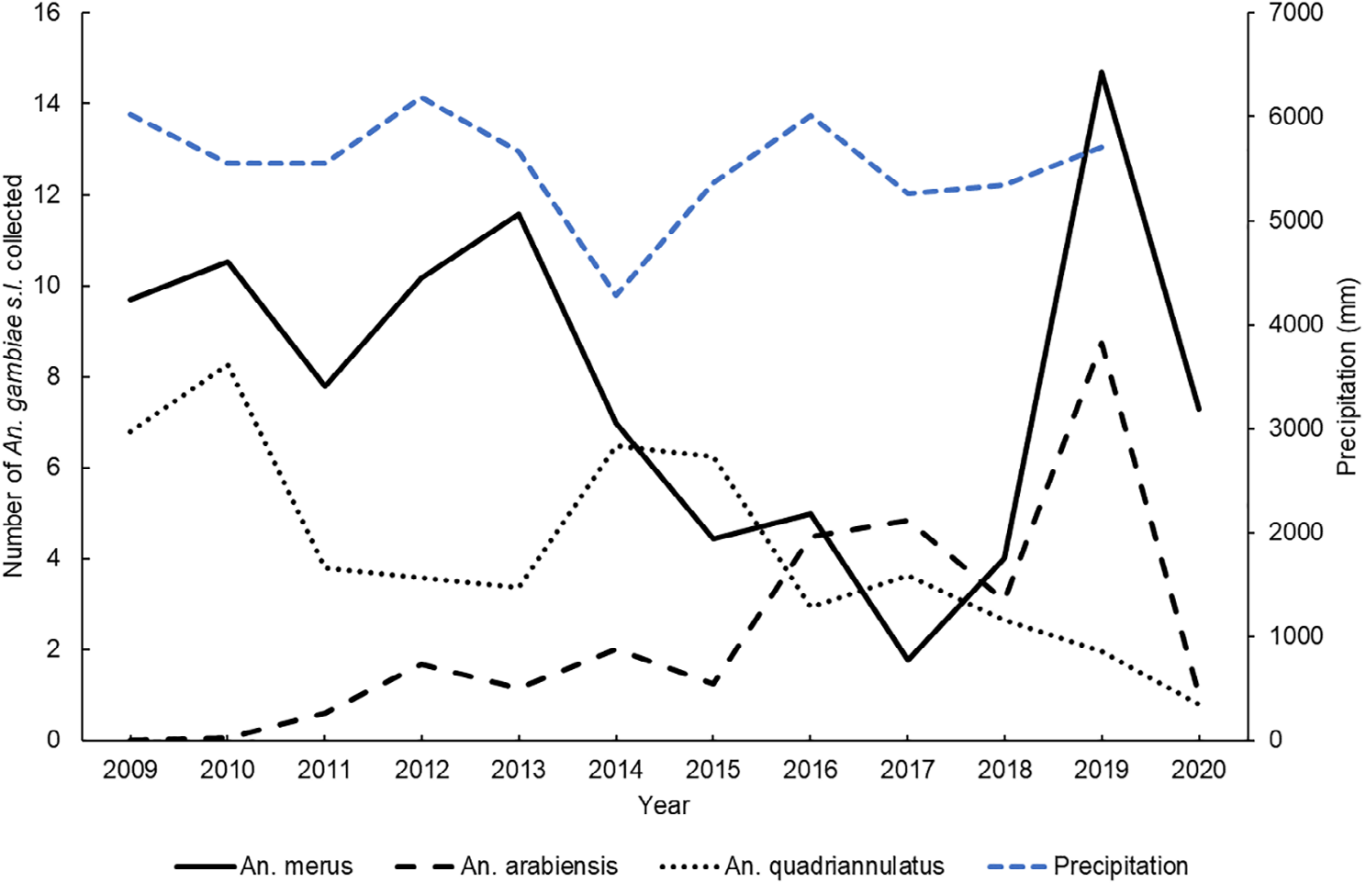
Average number of *Anopheles arabiensis*, *An. merus* and *An. quadriannulatus* captured annually per day from 2009 to 2021 in the Ehlanzeni district, Mpumalanga province, South Africa. The blue dotted line indicates the total annual precipitation in mm across the study period.

*Anopheles* relative abundance fluctuated over the 13‐year period for all three species (Figure 3). *Anopheles merus* was the most abundant species for the majority of the 13‐year surveillance period, with reduced abundance only during the period 2015 to 2018 (Figure 3). *Anopheles arabiensis* was present in lower numbers until 2015, then increased to become the dominant member of the *An. gambiae* complex in 2017 (Figure 3). The non‐vector *An. quadriannulatus* was more abundant than *An. arabiensis* until 2015, after which it became less abundant than *An. arabiensis* (Figure 3). In 2018, all three species were present in lower numbers (Figure 3). From 2019 to 2020, *An. merus* was the dominant species, and *An. quadriannulatus* numbers continued to decline (Figure 3).

### Vector abundance correlated with climatic parameters

*Anopheles arabiensis* showed a significant negative correlation with the average monthly temperature in Bushbuckridge (*r*_*s*_ = −0.7, *p* = 0.03). *Anopheles arabiensis* at Block A in Nkomazi showed a significant positive correlation with temperature on the day of collection (*r*_*s*_ = 0.17, *p* = 0.02), whereas *An. quadriannulatus* showed a significant negative correlation with temperature on the day of collection (*r*_*s*_ = −0.17, *p* = 0.02). All other climatic factors, including precipitation, were not correlated with vector abundance, regardless of species (*p* > 0.05 in all instances).

### Proportion of mosquitoes collected per collection method

The majority of samples were collected as larvae i.e. 90% of *An. arabiensis* collected were collected as larvae, while 85% of *An. merus* and 93% of *An. quadriannulatus* were collected as larvae.

**FIGURE 4:**
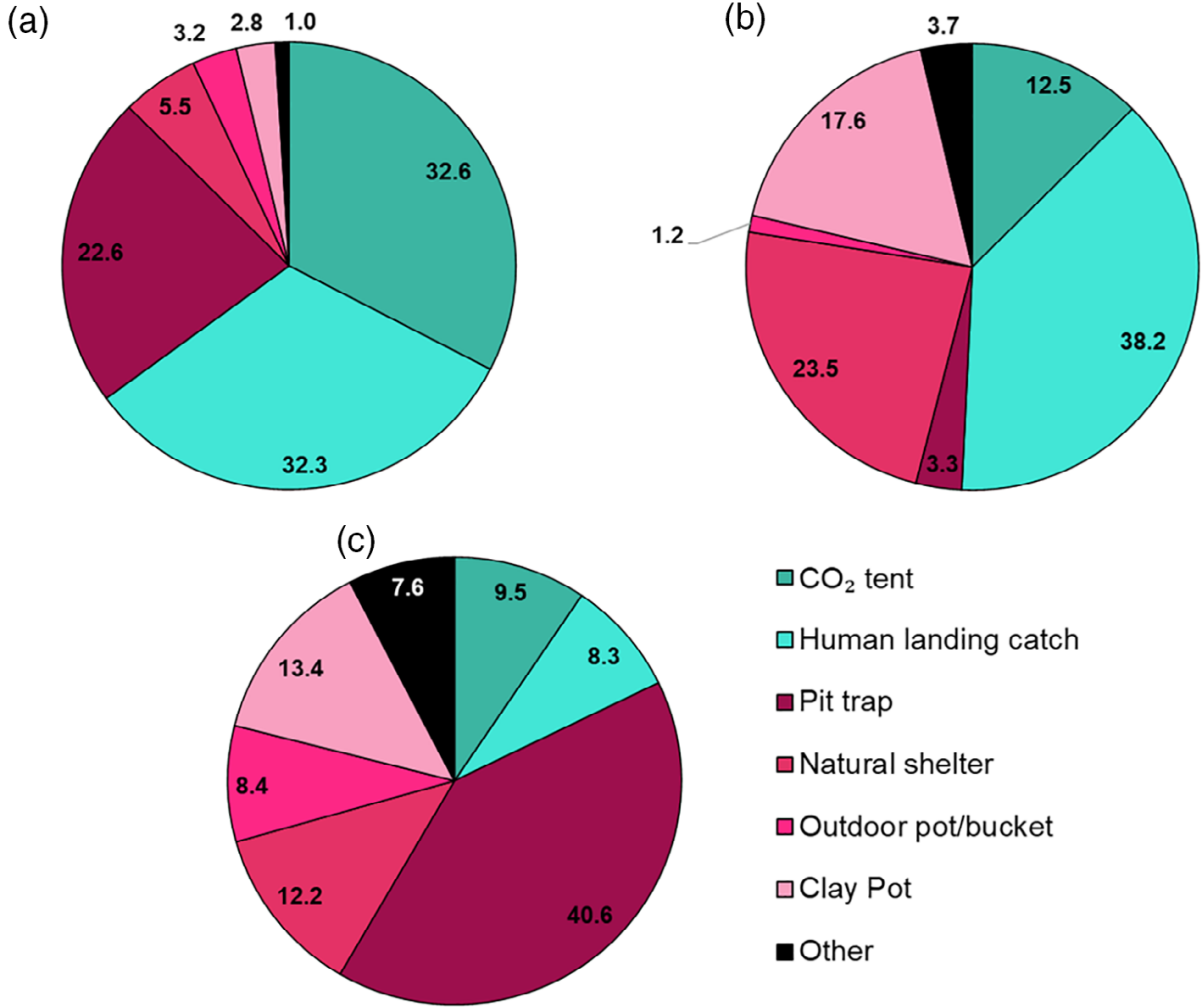
Number of *Anopheles gambiae s.l*. adults captured per day for each collection method used in the Ehlanzeni district, Mpumalanga province, South Africa, from 2009 to 2021. (a) *An. arabiensis* (b) *An. merus* and (c) *An. quadriannulatus*. Shades of cyan indicate catches of active mosquitoes and shades of magenta indicate passive catches. Numbers on the chart represent percentages.

Pit traps were the most effective method for capturing adult *An. quadriannulatus* while human landing catches were the most successful for capturing adult *An. arabiensis* (Figure 4). All three species were captured from CO_2_ tents, and this was the most effective method for capturing *An. merus*. Clay pots were effective for capturing *An. arabiensis* and *An. quadriannulatus*, but not *An. merus* (Figure 4). Other collection methods, including disused tyres, traps, indoor pots and pit toilets, were generally ineffective at capturing *An. gambiae s.l*. adults (Figure 4).

### Frequency of collection method usage across the sampling period

**FIGURE 5:**
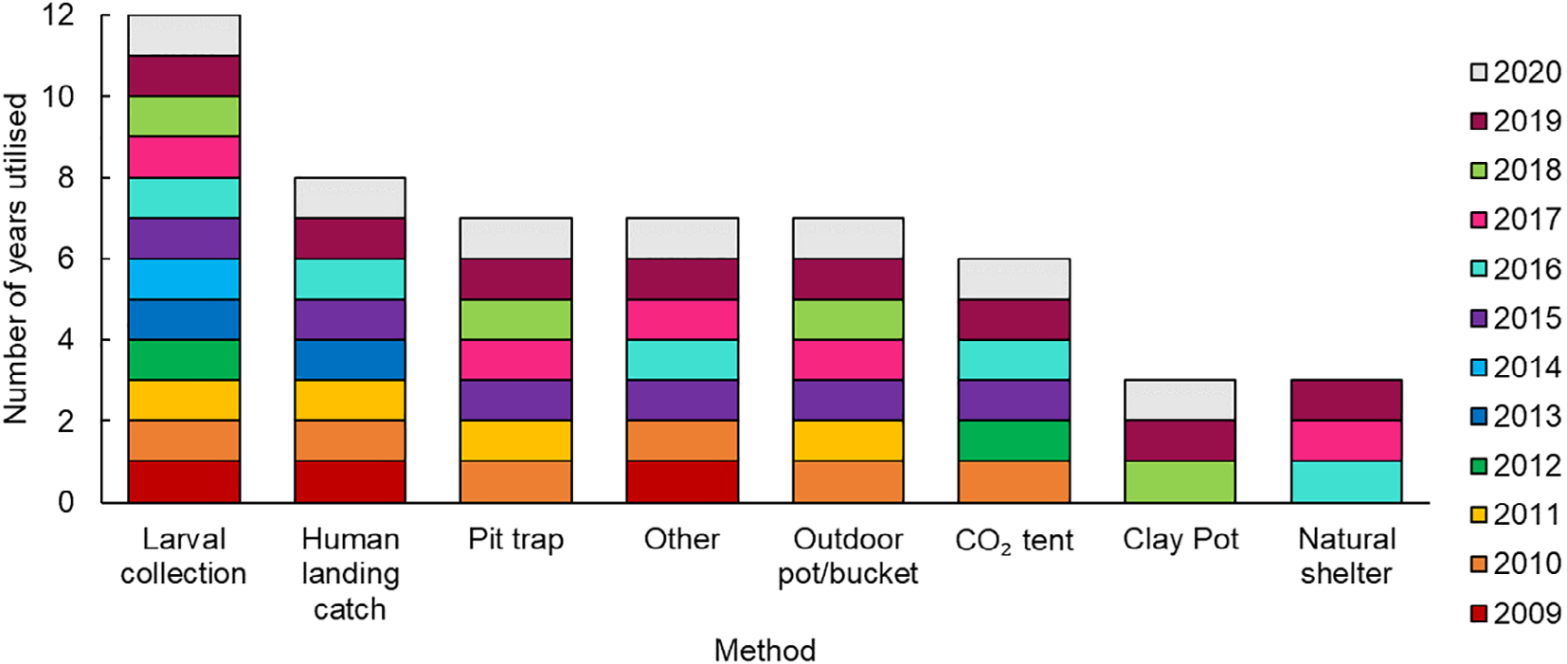
Frequency of different collection methods that were employed across the sampling period. Other methods include collections from dongas, indoor pots, pit toilets, window traps and disused tyres.

The frequency of the collection methods that were employed across the sampling period was not equal. Larval collections were the most consistent method used overall, while human landing catches were the most consistent method used to collect adult mosquitoes. Outdoor pots/buckets were used the least (Figure 5). With the exception of the clay pots, all other adult collection methods were used sporadically throughout the sampling period (Figure 5). The clay pots were introduced in 2018 and used consistently until the end of the sampling period (Figure 5).

### Species diversity by surveillance locality

A total of 71 sites were sampled across three municipal areas, viz. Nkomazi (*n* = 40), Bushbuckridge (*n* = 19) and Mbombela (*n* = 12) from 2009 to 2021.

Nkomazi had a mean species richness (R) of 2.4 ± 0.75 (rounded down to represent a species richness of 2). The mean Shannon‐Weiner Diversity index (H) for Nkomazi was 0.64 ± 0.36, while the mean Simpson's Diversity index (D) was 0.61 ± 0.23. The mean Pielou's Evenness index (J) for Nkomazi was 0.66 ± 0.35.

Mbombela had a mean species richness of 1.92 ± 0.79 (rounded up to represent a species richness of 2). The mean Shannon‐Weiner Diversity index for Mbombela was 0.40 ± 0.38, while the mean Simpson's Diversity index was 0.75 ± 0.24. The mean Pielou's Evenness index for Mbombela was 0.47 ± 0.41.

Bushbuckridge had a mean species richness of 1.26 ± 0.45 (rounded down to represent a species richness of 1). The mean Shannon‐Weiner Diversity index for Bushbuckridge was 0.12 ± 0.23, while the mean Simpson's Diversity index was 0.92 ± 0.16. The mean Pielou's Evenness index for Bushbuckridge was 0.18 ± 0.34.

**FIGURE 6:**
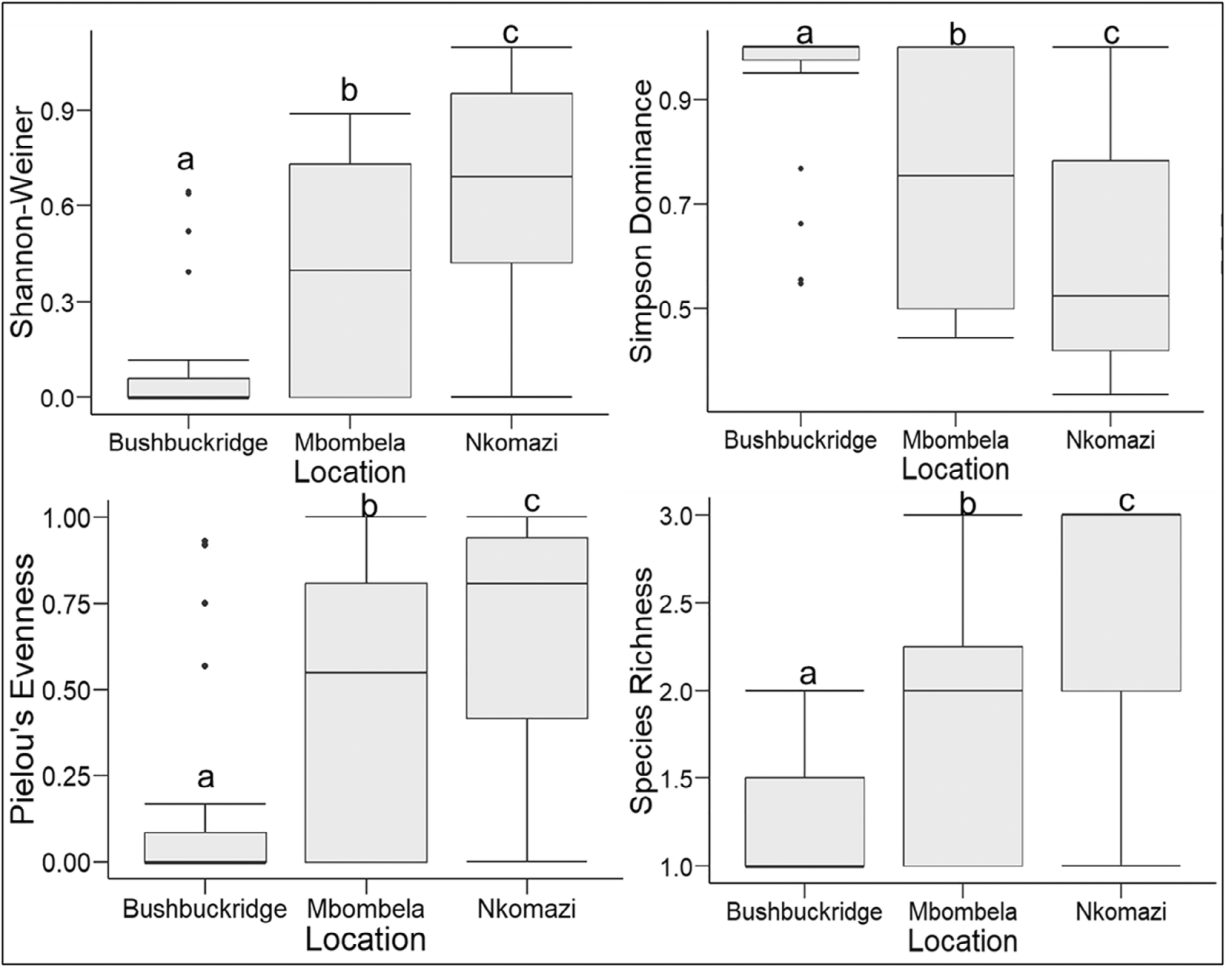
Alpha diversity of *Anopheles gambiae* complex species composition in the Ehlanzeni district in the three municipalities within the greater Ehlanzeni district, Mpumalanga province, South Africa, from 2009 to 2021. (a) species richness (b) Pielou's evenness index (c) Shannon‐Weiner diversity index and (d) Simpson Diversity index. The mean is indicated by an ‘x’ for each box.

The data for each of the indices were not normally distributed (W_D_ = 0.83, *p* = 2.092e−07; W_H_ = 0.85, *p* = 1.112e−06; W_J_ = 0.81, *p* = 3.215e−08 and W_R_ = 0.78, *p* = 6.99e−09). A Kruskal–Wallis test showed significant differences between districts for the Shannon‐Weiner Diversity Index (H: χ^2^[2, *n* = 71] = 22.94, *p* = 1.043e−05), the Simpson's Diversity Index (D: χ^2^[2, *n* = 71] = 21.55, *p* = 2.09e−05), the Pielou's Evenness index (J: χ^2^[2, *n* = 71] = 17.99, *p* = 0.00012) and species richness (R: χ^2^[2, *n* = 71] = 24.61, *p* = 4.534e−06) (Figure 6).

## DISCUSSION

This study was a retrospective examination of an existing entomological surveillance database, rather than a study of the historical bionomics of the Mpumalanga province. Several inferences on the historic trends in vector species composition, collection methodology and environmental effects could be made from the database that will aid South Africa's malaria elimination agenda. While no significant differences were present in sampling effort across the sampling period, there were a number of limitations in the data collection process, especially concerning the collection methodology. Addressing these limitations will strengthen the database going forward.

The database suggests that historically *An. merus* is the most abundant member of the *An. gambiae* complex in Mpumalanga province. This contrasts with KwaZulu‐Natal province, where *An. arabiensis* is the predominant member of the *An. gambiae* complex (Dahan‐Moss et al., 2020; Dandalo et al., 2017). A review of the anopheline diversity in Limpopo province showed that *An. merus* was the least prevalent member of the *An. gambiae* complex (Braack et al., 2020). This shows the heterogeneity in the distribution of anophelines in these study sites, highlighting the importance of understanding provincial malaria vector bionomics.

The dominance of *An. merus* in the Ehlanzeni district is congruent with previous surveillance data. Collections in the region between 1997 and 1998 showed that *An. merus* was the dominant species (56%), with *An. quadriannulatus* at a prevalence of 30.4% and *An. arabiensis* at 13.8% (Govere et al., 2000). Surveillance in the Ehlanzeni district between 2005 and 2014 indicated that *An. merus* made up 64% of the collections (Mbokazi et al., 2018).

*Anopheles merus* sampling abundance shows seasonality. Most specimens of this species were collected in spring, with the numbers declining to their lowest levels in winter. By contrast, most *An. arabiensis* specimens were collected in autumn, followed by summer, spring and winter seasons. For *An. quadriannulatus*, the greatest numbers were collected in spring, followed by autumn, summer and winter. Although this represents absolute numbers, it is worth noting that when examining the percentage distribution, *An. arabiensis* only constituted 8.8% of the spring total, but 13.8% of the winter total. Therefore, although as with both the other species, the total captures were lowest in winter, *An. arabiensis* had a greater proportional representation in winter than in spring. Autumn was the most evenly represented season, where the species proportions were approximately a third each and *An. merus* was at the lowest proportional representation. These differences in seasonality suggest either competition between the species (Ewing et al., 2019), or variation in ecological adaptation whereby *An. arabiensis* are better adapted to drier climatic conditions.

From the information available in the database, in general, there was no correlation with annual climatic parameters. However, this does not mean that there is no association between climate and vector abundance in the province. When examining rainfall in Nkomazi at Block A on a daily level, the correlation between climate and vector abundance increases and is significant at the 95% confidence interval. Several other studies found a significant correlation between vector abundance and climatic parameters at a site‐specific level on a monthly basis (Govere et al., 2000; Konwar et al., 2021; Manyi et al., 2015; Mosha & Mutero, 1982; Simon‐Oke & Olofintoye, 2015). This suggests that there may be significant correlations when examining abundance and rainfall/ temperature at a daily or weekly level. It is possible that there may be unexamined effects of microclimate on vector abundance. For instance, microclimatic variation has been demonstrated to affect the biting behaviour of *An. arabiensis* (Ngowo et al., 2017). As such, climatic variation may play a complex role in species abundance, but may not be detected on the scale of climate data available.

There is a notable climate anomaly though. In 2017, the numbers of *An. arabiensis* exceeded that of *An. merus* and *An. quadriannulatus* for the only time during the period under review. During 2017, the average daily rainfall in the province was higher than the average daily rainfall from 2007 to 2016 (NASA Langley Research Center Atmospheric Science Data Center, 2022). The higher rainfall may have hindered the population growth of *An. merus* and *An. quadriannulatus* by flushing out larval habitats (Paaijmans et al., 2007). Prolonged rains may also have diluted the salinity levels of water bodies primarily used by *An. merus*, thus enabling *An. arabiensis* to utilise these habitats. It is also not coincidental that 2017 marked South Africa's highest number of malaria cases since the epidemic of 2000 (Maharaj et al., 2019). Several studies have shown that rainfall is a significant driver of malaria transmission (Abiodun et al., 2018; Adeola et al., 2017; Adeola, Botai, et al., 2019; Adeola, Ncongwane, et al., 2019). This highlights the fact that climate's effect on the intensity of malaria transmission cannot be ignored.

The analysis of the collection methods was also informative. The relative efficiencies of the adult collection methods can be used to infer some mosquito behavioural characteristics. The dominant species, *An. merus*, were primarily collected by human landing catches, pit traps and CO_2_ tent catches at approximately equal ratios. These were also the most successful methods for collecting *An. quadriannulatus*, although pit traps were more successful than human landing catches, which in turn was more successful than CO_2_ tent catches. By contrast, there was more variability in the *An. arabiensis* catches. Human landing catches, however, were the most efficient mechanism for sampling this species. This suggests that although all three species engaged in human seeking, *An. arabiensis* was the most anthropophilic. Outdoor‐placed clay pots are a successful method for sampling *An. arabiensis* in northern KwaZulu‐Natal province (Burke et al., 2017; Dandalo et al., 2017; Munhenga et al., 2022), but their deployment was less successful in Mpumalanga. It is worth noting that clay pot collections made up a more substantial portion of *An. arabiensis* than the other two species. The results also suggest tailored methods to collect species of interest on a regional basis. For example, when trying to incriminate *An. arabiensis* as a potential vector, pit traps and natural shelters are not suitable trapping methods. Rather, human landing catches would be more efficient in collecting *An. arabiensis*. These are crucial considerations for the optimisation of the control programme in Mpumalanga.

There were several limitations identified during the analysis of the collection methodology. The number of traps, sampling hours and sampling personnel were not captured during data collection. Additionally, different collection methods were not employed with equal frequency across the sampling period. Finally, several different sampling sites were used across the sampling period, with no clear indication of whether the same sampling sites were used or whether sites were randomised. While there were no significant differences in sampling effort across the sampling period, standardising the collection methodology may strengthen the database and allow for more powerful analyses to be performed in future.

We suggest a standardised metadata template for collection, outlining the necessary data to capture when collecting field samples (Figure S2). Importantly, detailed locality data with GPS coordinates, trap number, number of sampling personnel and sampling hours should be added to the current body of data collected from the field. Further, employing the three best‐performing collections methods (i.e., larval collections, pit traps and CO_2_ tents) exclusively would be most suitable to standardise the data collection process. We also encourage sampling from the same localities as far as possible. Challenges arise when streams in certain areas dry up. To address this, permanent sampling sites can be established in localised areas such as Vlakbult or Masibekela, with collections carried out at micro‐sampling sites within those areas.

The Nkomazi Municipality had higher diversity and evenness indices than the other two municipalities, indicating that the diversity of *An. merus*, *An. arabiensis* and *An. quadriannulatus* in this district is comparatively high and that all three species are more or less evenly distributed, as opposed to Mbombela and Bushbuckridge where the diversity was low, and the three species were not evenly distributed. This may be because Mbombela and Bushbuckridge had a notably lower number of sampling sites compared to Nkomazi. Further, regular sampling in Mbombela only started in 2015 and for Bushbuckridge in 2014. All three species occurred in all three municipal districts, but did not necessarily all occur together at the same time. Previous studies also found high diversity and species evenness at Nkomazi (Govere et al., 2000; Mbokazi et al., 2018; Ramalwa et al., 2014). Mbokazi et al. (2018) study similarly found that species diversity was low for the Mbombela and Bushbuckridge municipalities, as well as a low species evenness for Bushbuckridge. Species richness for Mbombela was however found to be fairly evenly distributed in their study. Significant differences existed between the three sites for both the Shannon‐Weiner and Simpson diversity index, which takes into consideration both species richness and evenness. Crucially, these patterns are inverted (e.g. Nkomazi has the highest Shannon‐Weiner index and the lowest Simpson index), as expected. This indicates that, even with the limited amount of data, the distribution that can be determined from the database is valid.

This review has provided valuable insights into historical trends of *Anopheles gambiae* complex species composition, collection trends and environmental effects. Additionally, this review has identified a number of limitations in the data collection process. Going forward, what this review also presents are considerations on how to improve and strengthen the database for future analyses. This includes using permanent sentinel sampling sites, employing the three best‐performing collection methods and collecting additional information during the collection process. Additionally, recording climatic variables at the sampling site level, as opposed to the regional level, is essential for understanding the relationship between climate and vector abundance. These all, however, are limited by logistical factors. Despite this, if such factors could be employed to strengthen this database, it would make a greater impact on South Africa's elimination agenda.

In conclusion, a substantial amount of data could be extracted from the VCRL database. These data provide information on the population fluctuations of the *An. gambiae* complex in Mpumalanga province. It enables some inferences concerning adaptability to the climate variables, rainfall and temperature, and they indicate seasonal abundance and geographical distribution by species. Importantly, these data show the efficacy of various collection methods for the capture of *An. gambiae* complex mosquitoes in the province. This information also highlights the need for deeper investigations into the role of climate, and microclimate in particular, on vector biology. This information can be used to tailor vector control in this province in terms of timing of interventions and also indicates risk and receptivity for malaria based on vector occurrence and distribution—an important indicator for malaria elimination certification.

## AUTHOR CONTRIBUTIONS

**Kayla P. Noeth:** Conceptualization; investigation; writing – original draft; formal analysis; project administration; writing – review and editing. **Maria L. Kaiser:** Writing – review and editing; investigation; resources; data curation. **Thabo Mashatola:** Writing – review and editing; investigation; resources. **Yael L. Dahan‐Moss:** Data curation; writing – review and editing. **P. Avhatakali Matamba:** Formal analysis; writing – review and editing. **Belinda Spillings:** Formal analysis; writing – review and editing. **Riann Christian:** Writing – review and editing; formal analysis; data curation. **Erika Erlank:** Investigation; resources; formal analysis; writing – review and editing. **B. Power Tshikae:** Writing – review and editing; investigation; resources. **Eunice Jamesboy:** Writing – review and editing; formal analysis. **Silindile Sibambo:** Investigation; resources; writing – review and editing. **Busisiwe G. Nkosi:** Investigation; writing – review and editing; resources. **Brian T. Silawu:** Investigation; writing – review and editing; resources. **Lazarus J. Mkhabela:** Investigation; writing – review and editing; resources. **Fanuel S. Ndlovu:** Investigation; writing – review and editing; resources. **Thembekile P. Mgwenya:** Investigation; writing – review and editing; resources. **Maureen Coetzee:** Funding acquisition; writing – review and editing; supervision. **Basil D. Brooke:** Funding acquisition; writing – review and editing; supervision. **Lizette L. Koekemoer:** Writing – review and editing; supervision; funding acquisition. **Givemore Munhenga:** Funding acquisition; writing – review and editing; supervision. **Shüné V. Oliver:** Funding acquisition; supervision; project administration; conceptualization; writing – review and editing; formal analysis.

## FUNDING INFORMATION

This work was funded by the following agencies: Bill and Melinda Gates Foundation grant (Grant number OPP1210314) and partly funded by the International Atomic Energy Agency (IAEA) under their Technical Cooperation Programme (SAF 5014/5017); the National Research Foundation of South Africa (Grant numbers 119765 and 107428 to G. Munhenga and SRUG190313423259, SRUG2204133394, and CSRP210405592233 to S. V. Oliver and Department of Science and Technology–National Research Foundation Research Chairs Initiative grant [64763]); Joint Global Health Trials (MR/L00433X/1); the South African Medical Research Council Self‐Initiated Research Grant programme; the Female Academic Leadership Fellowship; and the National Health Laboratory Services Research Trust award.

## CONFLICT OF INTEREST STATEMENT

The authors declare no conflicts of interest.

## Supporting information


**Figure S1.** Equations used to calculate diversity indices.

**Figure S2.** Proposed metadata template to be used with example information to be collected during future surveillance activities.

## Data Availability

Data are not publicly available.
