# Supplementary figures and images for: A review of historical trends in Anopheles gambiae Giles (Diptera: Culicidae) complex composition, collection trends and environmental effects from 2009 to 2021 in Mpumalanga province, South Africa
Source: Med Vet Entomol. 2024 Sep 5;39(1):103–14. doi: 10.1111/mve.12761 (PMC11793130; doi:10.1111/mve.12761)

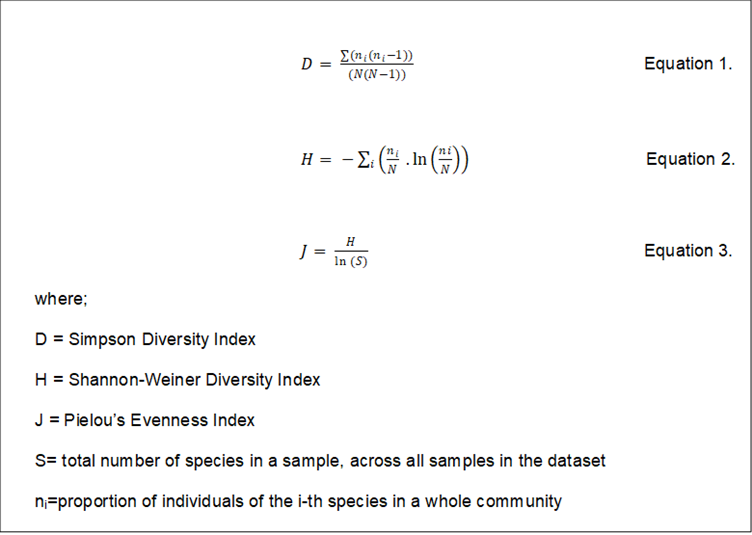

Supplement: Supplementary file 1 — Figure S1. Equations used to calculate diversity indices. [file MVE-39-103-s002.tif]

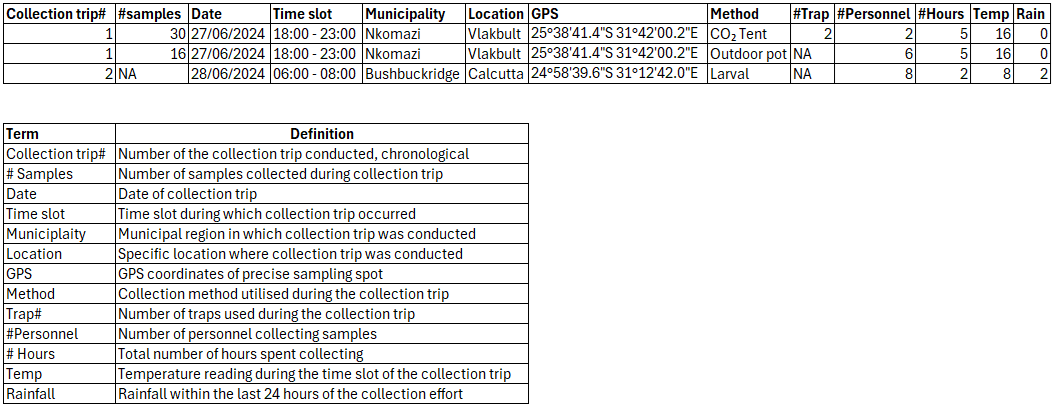

Supplement: Supplementary file 2 — Figure S2. Proposed metadata template to be used with example information to be collected during future surveillance activities. [file MVE-39-103-s001.tif]
